# Decoding m^6^A readers: roles of YTHDF proteins in leukemogenesis and cancer immunity

**DOI:** 10.3389/fimmu.2025.1692608

**Published:** 2025-12-01

**Authors:** Chunhong Li, Xi Chen, Shanrui Pu, Kun Lian, Lihua Li, Xiulin Jiang, Qiang Wang

**Affiliations:** 1 Department of Oncology, Suining Central Hospital, Suining, Sichuan, China; 2 NHC Key Laboratory of Drug Addiction Medicine, Kunming Medical University, Kunming, China; 3 School of Biosciences, University of Birmingham, Birmingham, United Kingdom; 4 Department of Systems Biology, City of Hope Comprehensive Cancer Center Biomedical Research Center, Monrovia, CA, United States; 5 Department of Gastrointestinal Surgical Unit, Suining Central Hospital, Suining, Sichuan, China

**Keywords:** YTHDF, m^6^A, hematopoietic stem cells, leukemia, cancer immunity, immune checkpoint therapy, epitranscriptomic regulation

## Abstract

N^6^-methyladenosine (m^6^A) is the most prevalent internal modification in eukaryotic mRNA and plays critical roles in post-transcriptional gene regulation. Among the m^6^A regulators, YTH domain family proteins (YTHDF1, YTHDF2, and YTHDF3) act as major “readers” that interpret m^6^A marks and dictate the fate of modified transcripts through coordinated control of mRNA translation, stability, and decay. Recent advances have uncovered multifaceted roles of YTHDF proteins in both physiological hematopoiesis and pathological leukemogenesis. YTHDF2 is essential for hematopoietic stem cell (HSC) self-renewal and lineage commitment by selectively degrading transcripts that constrain stemness, while dysregulated YTHDF activity contributes to leukemic stem cell maintenance, metabolic adaptation, and therapy resistance. In parallel, YTHDF1 and YTHDF3 have been implicated in shaping the leukemic transcriptome and cooperating with oncogenic signaling pathways to promote malignant transformation. Beyond intrinsic leukemic functions, accumulating evidence highlights the impact of YTHDF proteins on tumor immunity. By modulating dendritic cell antigen presentation, T cell activation, and immune checkpoint expression, YTHDF proteins orchestrate the tumor immune microenvironment and influence anti-tumor immunity. These discoveries not only provide mechanistic insight into how m^6^A readers govern hematopoietic and immune regulation, but also open new therapeutic avenues. Pharmacological manipulation of YTHDF activity holds promise to selectively eradicate leukemic stem cells, enhance immune surveillance, and improve responses to conventional and immune-based therapies. In this review, we summarize the latest progress in understanding the functional roles and molecular mechanisms of YTHDF proteins in normal hematopoiesis, leukemogenesis, and cancer immunity, and discuss emerging strategies for targeting m^6^A readers in hematologic malignancies and immunotherapy.

## 1 Introduction

m^6^A is the most abundant and evolutionarily conserved internal modification in eukaryotic mRNA, occupying a central position in post-transcriptional gene regulation (1, 2). As a dynamic and reversible epitranscriptomic modification, m^6^A precisely modulates multiple aspects of RNA metabolism-including splicing, nuclear export, stability, translation efficiency, and decay-through the coordinated actions of “writers” (methyltransferases), “erasers” (demethylases), and “readers” (binding proteins) (3–6). Among these regulators, reader proteins play a pivotal role by directly recognizing m^6^A-modified sites and determining the fate of target transcripts, thereby serving as the critical link between m^6^A marks and functional outcomes (7–9). YTHDF1, YTHDF2, and YTHDF3 are the most extensively studied m^6^A readers (10–12). They share a conserved YTH domain that specifically binds to m^6^A-modified RNA, yet each exerts distinct and partially cooperative functions. YTHDF1 primarily enhances the translation of its targets (13–15), YTHDF2 recruits deadenylase complexes and exoribonucleases to accelerate mRNA decay (16), and YTHDF3 acts as a modulator that coordinates the actions of YTHDF1 and YTHDF2 to maintain transcript homeostasis (17). This division of labor enables YTHDF proteins to play indispensable roles in maintaining cellular equilibrium and orchestrating responses to environmental changes.

Accumulating evidence highlights the unique and critical functions of YTHDF proteins in hematopoiesis and immunity (18). In normal hematopoiesis, YTHDF2 is essential for balancing self-renewal and differentiation of HSCs, with dysregulation leading to hematologic abnormalities and disease (19). Under pathological conditions, YTHDF proteins regulate the survival, metabolism, and signaling of leukemic stem cells (LSCs), thereby driving leukemia initiation and progression (20). Leukemia development is driven not only by cell-intrinsic genetic and epigenetic alterations that sustain LSC self-renewal and malignant progression, but also by profound remodeling of the immune microenvironment. These two processes are highly intertwined. On the one hand, leukemic cells actively reshape the immune landscape by impairing antigen presentation, suppressing cytotoxic T-cell responses, and promoting immunosuppressive populations such as MDSCs and regulatory T cells. On the other hand, the immune microenvironment reciprocally influences leukemic evolution by exerting selective pressure, modulating therapy responses, and contributing to immune escape. Within this bidirectional framework, m6A readers of the YTHDF family act at the intersection of leukemic signaling and immune regulation. YTHDF2 controls LSC maintenance through selective mRNA decay, while also affecting dendritic cell function and T-cell activation, thereby linking leukemic intrinsic programs to immune extrinsic regulation. YTHDF1 and YTHDF3 further integrate oncogenic transcriptional networks with immune checkpoint pathways and antigen-presentation machinery. By coordinating mRNA fate in both leukemic cells and immune cells, YTHDF proteins provide a unifying molecular mechanism connecting leukemogenesis with tumor immunology. This conceptual framework not only clarifies how YTHDF-mediated post-transcriptional regulation simultaneously orchestrates leukemic progression and immune microenvironment remodeling, but also highlights why targeting m6A readers may yield dual benefits-direct eradication of LSCs and enhancement of anti-tumor immunity.

Since the discovery of m^6^A RNA modification, numerous reviews have summarized the biological functions of individual YTHDF proteins. However, most of these studies have focused on specific members or particular tumor types, providing only a fragmented understanding of their roles. In contrast, our review offers a systematic and integrative summary of the entire YTHDF family, highlighting their distinct and coordinated functions in normal hematopoiesis, leukemogenesis, and tumor immune regulation. Rather than a general overview of m^6^A signaling in cancer, we emphasize the mechanistic diversity and context-dependent effects of YTHDF1, YTHDF2, and YTHDF3, aiming to provide a more comprehensive perspective that links their molecular functions to hematologic and immune homeostasis as well as malignant transformation.

## 2 Structural and functional features of YTHDF proteins

**Figure 1:**
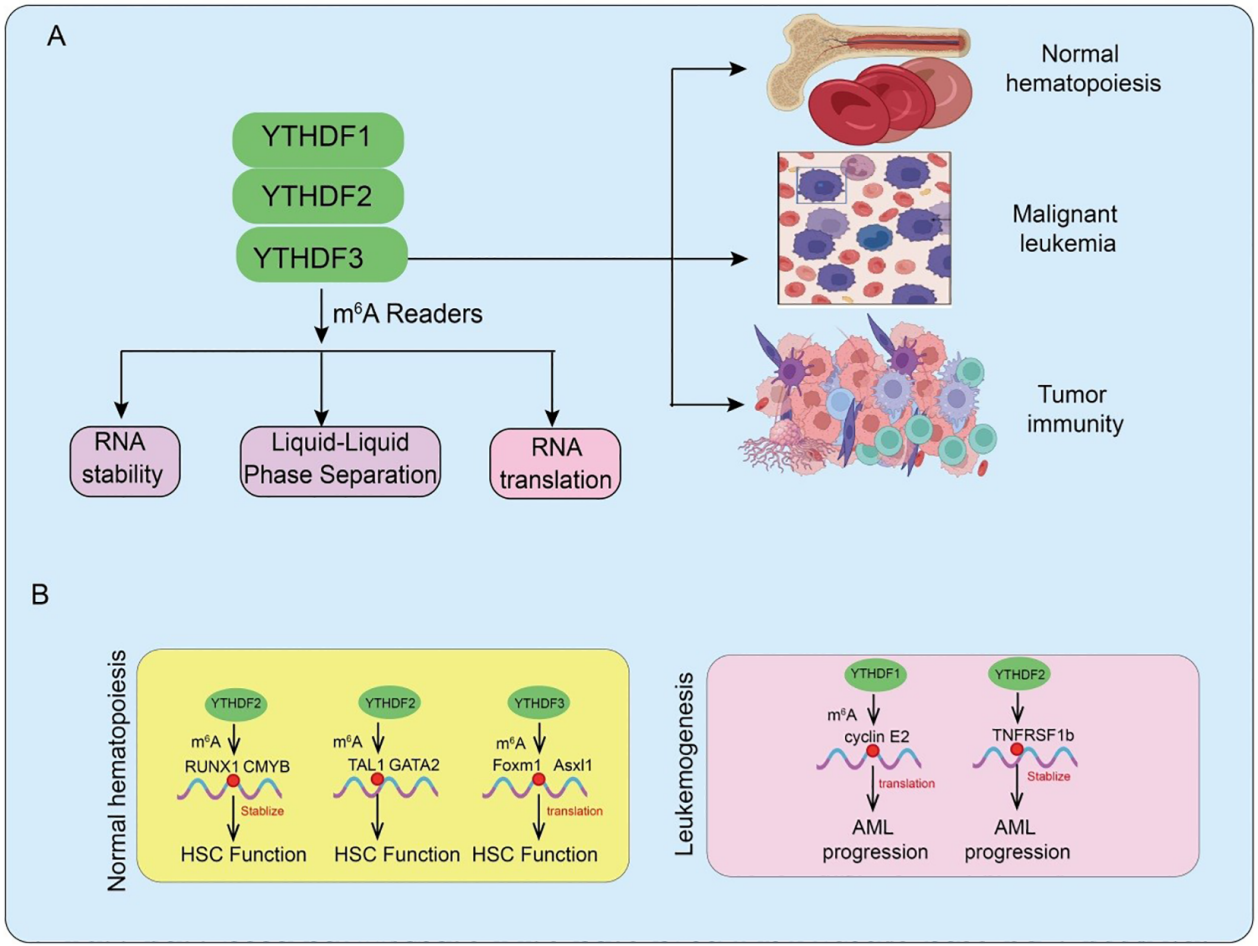
**(A, B)** the biological functional and distinct roles of YTHDF proteins in normal hematopoiesis and leukemogenesis. The YTHDF family (YTHDF1, YTHDF2, and YTHDF3) shares a conserved C-terminal YTH domain, which specifically recognizes and binds m^6^A-modified RNA, and an N-terminal LCD enriched in intrinsically disordered sequences. Despite their structural similarity, YTHDF proteins play distinct yet coordinated roles in hematopoiesis, leukemia, and tumor immune regulation, reflecting their context-dependent m^6^A-mediated regulatory functions. Under physiological conditions, YTHDF2 stabilizes m^6^A-modified transcripts such as RUNX1, CMYB, TAL1, and GATA2 to maintain HSC homeostasis, while YTHDF3 facilitates the translation of Foxm1 and Asxl1 mRNAs to support HSC function. In contrast, during leukemogenesis, YTHDF1 enhances the translation of cyclin E2, and YTHDF2 stabilizes TNFRSF1b transcripts, both promoting AML progression.

YTHDF1, YTHDF2, and YTHDF3 were the first cytoplasmic m^6^A readers to be identified, and their structural and functional features form the foundation of their diverse post-transcriptional regulatory roles (21–23). Each YTHDF protein is composed of two major modules: C-terminal YTH domain: Highly conserved (24), this domain recognizes and binds m^6^A-modified RNA with high specificity through an aromatic cage, thereby ensuring transcript-level targeting (25). N-terminal low-complexity domain (LCD): Enriched in intrinsically disordered sequences, this region facilitates protein–protein interactions and liquid–liquid phase separation (LLPS) (26–28). Such properties enable YTHDF proteins to localize to RNA metabolism-related granules, such as stress granules and P-bodies, and to dynamically participate in RNA regulation.Despite their structural similarity, early studies suggested that the three YTHDF proteins have partially distinct functions:YTHDF1: promotes ribosome recruitment and enhances mRNA translation efficiency (13). YTHDF2: recruits deadenylase complexes and RNA decay machinery to mediate rapid mRNA degradation.YTHDF3: functions as a coordinator, assisting YTHDF1 in translation and cooperating with YTHDF2 in degradation, thereby balancing translation and decay (17, 19). With the advent of high-throughput sequencing and single-cell technologies, the debate over whether YTHDF proteins act redundantly or exert specific functions has intensified: Redundancy hypothesis: Studies using knockout models revealed that deletion of a single YTHDF protein results in limited transcriptomic or phenotypic changes (27), whereas simultaneous loss of all three leads to pronounced mRNA instability and severe cellular dysfunction-supporting functional redundancy (23). Specificity hypothesis: Other studies demonstrated that YTHDF proteins display context-dependent roles. For instance, YTHDF2 is indispensable for HSC homeostasis, while YTHDF1 exerts unique functions in antigen presentation by immune cells (26). These findings argue for functional specificity under certain biological conditions. A more integrative perspective suggests that YTHDF proteins share overlapping binding profiles and potential redundancy at the molecular level, but their spatial expression patterns, interaction partners, and cellular contexts collectively determine their functional specificity. Thus, the YTHDF family may embody both redundancy and specificity, with their roles dynamically shaped by physiological and pathological states. This duality provides an important framework for interpreting their divergent functions in hematopoiesis, leukemia, and immune regulation (**Figure 1A**).

## 3 Mechanistic insights into the role of YTHDF in hematopoietic stem cells

The hematopoietic system is one of the most dynamic and regenerative tissues in the body, responsible for the continuous production of red blood cells, white blood cells, and platelets to sustain oxygen delivery, immune defense, and hemostasis (29, 30). Its core driving force is the HSC population, which is characterized by two fundamental properties: self-renewal and multilineage differentiation (31). On one hand, HSCs can maintain their stemness over the lifetime of an organism, thereby ensuring the long-term functionality of the hematopoietic system (32, 33). On the other hand, they can differentiate into various hematopoietic progenitors, which further develop into diverse mature blood lineages according to physiological needs (34, 35). The fate of HSCs is tightly regulated at multiple levels, including transcription factors, epigenetic modifications, signaling pathways, and the bone marrow microenvironment (36, 37). Among these regulators, post-transcriptional modifications such as m^6^A have emerged as crucial determinants of HSC self-renewal and lineage specification (38, 39). Proper maintenance of HSC function is essential for hematopoietic homeostasis, while dysregulation may result in bone marrow failure, immune deficiencies, and the development of hematologic malignancies such as leukemia (**Figure 1B**).

Current evidence indicates that YTHDF1 does not significantly affect HSC self-renewal or the maintenance of the hematopoietic system (40). In contrast, YTHDF2 plays a pivotal role in restricting the excessive expansion of HSCs and early progenitors, thereby preserving the balance of hematopoietic differentiation (19). Loss of YTHDF2 enhances HSC self-renewal and reconstitution capacity but results in a myeloid-biased differentiation and partial impairment of T-cell development. YTHDF2 is highly expressed in HSCs, where it selectively degrades transcripts that promote excessive proliferation or impair stemness maintenance, thus safeguarding the long-term self-renewal potential of HSCs (19). Mouse models have demonstrated that deletion of Ythdf2 causes HSC expansion but ultimately leads to stem cell exhaustion, impaired differentiation capacity, and hematopoietic imbalance (19). Mechanistically, YTHDF2 also restrains chronic activation of inflammatory pathways in HSCs by degrading m^6^A-modified inflammation-related transcripts. Loss of YTHDF2 results in progressive HSC exhaustion upon serial transplantation, manifested by myeloid skewing, loss of lymphoid potential, and long-term hematopoietic failure-particularly pronounced in aged mice. Experimental evidence shows that inflammatory stress induces YTHDF2 expression, which protects HSCs from inflammation-induced damage. Collectively, these findings identify YTHDF2 as a key suppressor of HSC inflammatory responses, underscoring the essential role of m^6^A regulation in sustaining long-term hematopoietic homeostasis (19).

Compared with YTHDF2, YTHDF3 has been less studied. Our group previously demonstrated that YTHDF3 is dispensable for steady-state hematopoiesis: Ythdf3 knockout mice remain viable with normal blood cell counts, bone marrow cellularity, and frequencies of HSCs and hematopoietic progenitor cells (HPCs). Moreover, HSC quiescence and apoptosis were unaffected, indicating that YTHDF3 is not essential for maintaining HSC/HPC homeostasis under basal conditions. However, under stress conditions, YTHDF3 becomes indispensable (41). Following transplantation or 5-fluorouracil (5-FU) treatment, loss of Ythdf3 led to reduced bone marrow cellularity, decreased LSK and HSC/MPP populations, and increased HSC apoptosis, accompanied by severe defects in hematopoietic reconstitution and long-term self-renewal. Consistently, other studies have shown that Ythdf3 deficiency significantly impairs HSC repopulating capacity, resembling the phenotype of Mettl3 deletion, both of which result from defective translation of Ccnd1. Mechanistically, YTHDF3 cooperates with METTL3 by recognizing and relaying m^6^A marks on the 5′UTR of Ccnd1 to promote its translation. Functional rescue experiments revealed that forced expression of Ccnd1 fully restored the defects of Ythdf3^−/−^ HSCs and partially compensated for the loss of Mettl3 (42). These results highlight the critical role of YTHDF3 in supporting HSC survival and self-renewal under stress conditions.

In comparison, the role of YTHDF1 in hematopoiesis remains less well defined. While current evidence suggests that YTHDF1 is not required for steady-state hematopoiesis, it may contribute to specific stages or lineage differentiation processes. By enhancing translation, YTHDF1 could support the increased protein synthesis demands of proliferating progenitors. Although studies remain limited, these findings suggest that YTHDF1 and YTHDF3 are not entirely redundant but instead exhibit context-dependent functions in the hematopoietic environment. Collectively, current research indicates that the m^6^A-YTHDF axis provides a highly efficient and flexible regulatory mechanism for hematopoiesis. Under steady-state conditions, YTHDF2 maintains stemness and prevents abnormal HSC expansion. During stress or differentiation (19), YTHDF1 and YTHDF3 functions may be activated to fine-tune translation and transcript turnover, enabling rapid adaptation to hematopoietic demands. The complementary and cooperative actions among YTHDF proteins ensure the dynamic equilibrium of the hematopoietic system under both homeostatic and stress conditions. These insights position YTHDF proteins not merely as passive executors of post-transcriptional modifications but as central regulators of hematopoietic fate decisions. Elucidating their roles will not only advance our understanding of normal hematopoiesis but also shed light on their dysregulation in leukemogenesis.

## 4 Function and mechanism of YTHDF in leukemia

The initiation and progression of leukemia are closely linked to aberrant epigenetic regulation (43–45). In recent years, mounting evidence has revealed that m^6^A modification and its reader proteins, the YTHDF family, are aberrantly exploited in leukemic cells and LSCs, thereby promoting malignant transformation, sustaining disease progression, and driving therapeutic resistance (40). YTHDF1 is overexpressed in AML, particularly enriched in LSCs. Loss of YTHDF1 markedly impairs the self-renewal, proliferation, and leukemogenic capacity of AML cells, while sparing normal hematopoiesis. Mechanistically, YTHDF1 promotes the translation of CCNE2 (Cyclin E2) in an m^6^A-dependent manner (40). Structure-based drug screening has identified the FDA-approved drug Tegaserod as a potential YTHDF1 inhibitor, which blocks its interaction with m^6^A-modified RNA, suppresses CCNE2 translation, reduces AML cell viability, and prolongs survival in patient-derived xenograft models (40). Similarly, CSRP1 is significantly upregulated in AML, where it promotes proliferation and glycolysis. METTL3 stabilizes CSRP1 mRNA through m^6^A modification, which is then recognized and bound by YTHDF1, further enhancing its expression (46). This METTL3/YTHDF1/CSRP1 axis drives AML progression and represents a potential therapeutic target.

AML is an aggressive clonal disorder characterized by differentiation arrest of HSCs and progenitors, resulting in the formation of LSCs (47–49). YTHDF2 is broadly upregulated in AML and is indispensable for disease initiation and maintenance. By promoting the degradation of multiple m^6^A-modified transcripts, including those regulating Tnfrsf2, YTHDF2 sustains LSC function. Loss of YTHDF2 stabilizes Tnfrsf2 mRNA, leading to apoptosis of LSCs (19). Importantly, YTHDF2 is dispensable for normal HSCs and its loss even enhances their regenerative capacity, highlighting its unique therapeutic potential: inhibition of YTHDF2 not only selectively eradicates LSCs but may also expand functional HSCs (19). Furthermore, YTHDF2 is aberrantly elevated in relapsed AML patients, where it binds to m^6^A-modified pre-miR-126, recruits AGO2, and promotes its maturation into the oncogenic miRNA miR-126, thereby enhancing leukemic proliferation and leukemogenesis (50). Functional rescue experiments showed that reintroduction of miR-126 counteracts the tumor-suppressive effects of YTHDF2 depletion, indicating that the YTHDF2/miR-126 axis represents a critical oncogenic pathway and a potential therapeutic vulnerability in AML (50). Recent studies have provided intriguing evidence that YTHDF2 exerts dual oncogenic roles in B-cell malignancies: enhancing energy metabolism and promoting immune evasion (51). Overexpression of YTHDF2 alone is sufficient to drive B-cell transformation and lymphomagenesis. Mechanistically, YTHDF2 functions as a bifunctional reader protein: on one hand, it binds m^5^C-modified transcripts and recruits PABPC1 to stabilize mRNAs and enhance ATP synthesis; on the other, it acts as an m^6^A reader to degrade immune-related mRNAs, thereby facilitating immune escape. Pharmacological inhibition of YTHDF2 suppresses malignant progression and improves the efficacy of CAR-T therapy in preclinical models (51).

By contrast, the role of YTHDF3 in AML remains poorly defined. To date, no direct functional evidence has been reported, likely because YTHDF3 primarily acts in cooperation with YTHDF1 and YTHDF2-enhancing mRNA translation or accelerating decay-rather than exerting strong independent effects (52). Its relatively lower expression in AML cells and patient samples, coupled with its non-essential role in LSC survival and self-renewal, may explain why YTHDF3 has received less attention. Taken together, YTHDF proteins exhibit a “double-edged sword” effect in leukemia: while they maintain homeostasis in normal hematopoiesis, under pathological conditions they are hijacked to support LSC survival, sustain malignant properties, and enable therapeutic resistance. A deeper understanding of these mechanisms will not only illuminate the epitranscriptomic basis of leukemogenesis but also provide a theoretical foundation for the development of therapeutic strategies selectively targeting the YTHDF–m^6^A regulatory axis.

## 5 The role and mechanistic basis of YTHDF in tumor immunity

**Figure 2:**
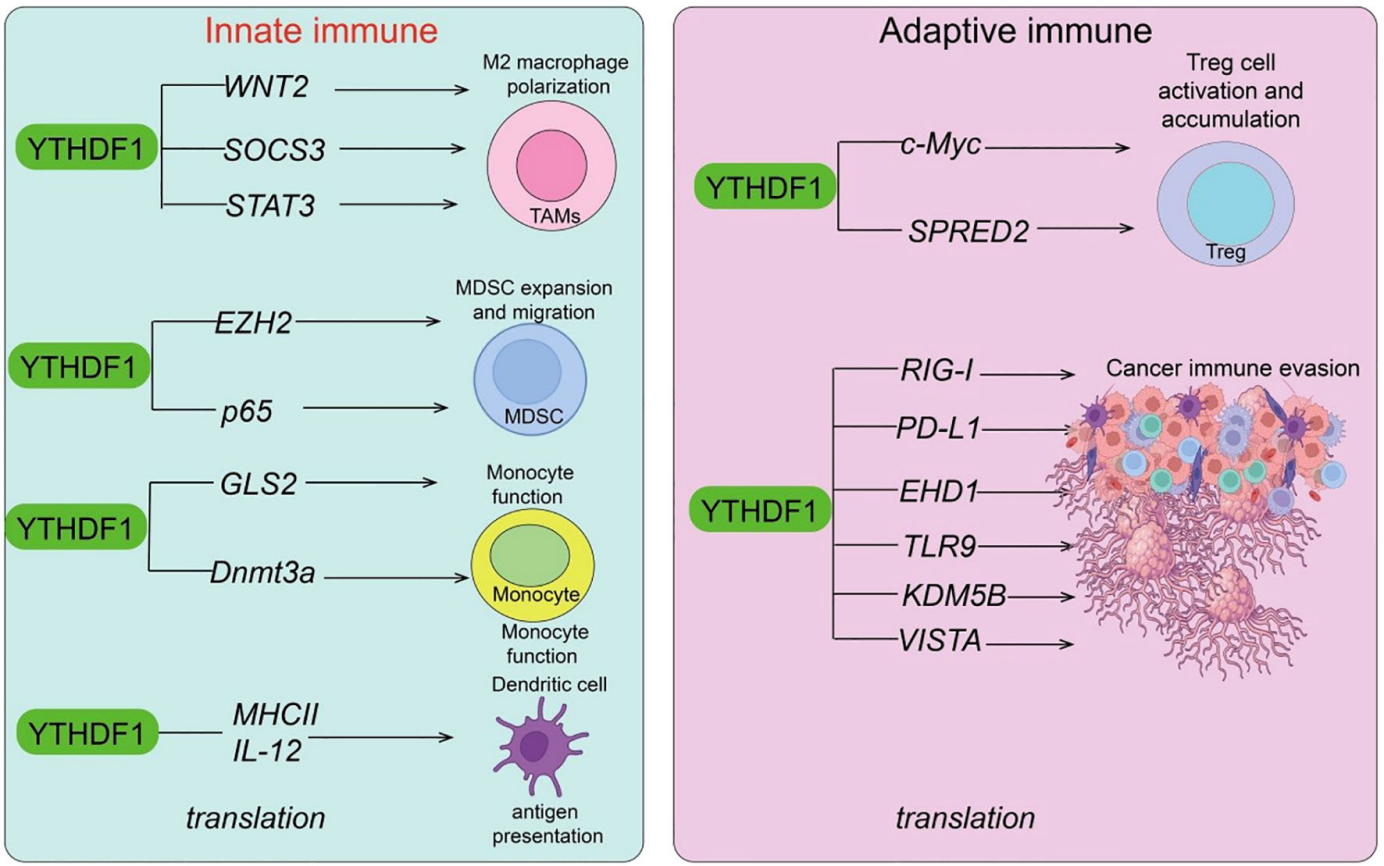
Roles of YTHDF1 in innate and adaptive immune regulation. In innate immunity, YTHDF1 promotes M2 macrophage polarization and tumor-associated macrophage (TAM) formation by regulating WNT2, SOCS3, and STAT3 translation. It facilitates myeloid-derived suppressor cell (MDSC) expansion and migration via EZH2 and p65, and modulates monocyte and dendritic cell (DC) function through GLS2, Dnmt3a, MHCII, and IL-12. In adaptive immunity, YTHDF1 enhances Treg activation and accumulation by upregulating c-Myc and SPRED2, and promotes cancer immune evasion by regulating the translation of RIG-I, PD-L1, EHD1, TLR9, KDM5B, and VISTA.

**Figure 3:**
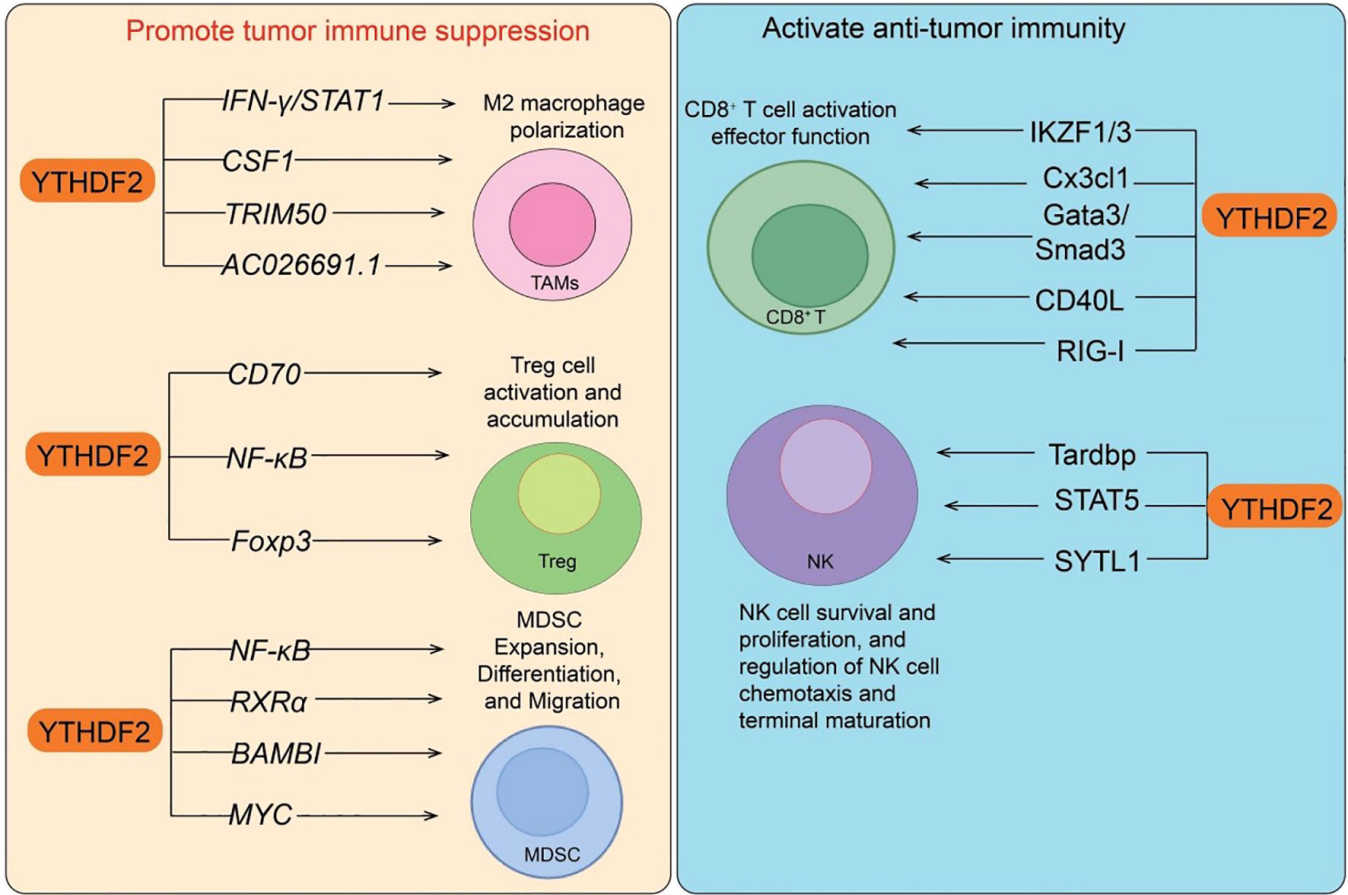
Dual roles of YTHDF2 in regulating tumor immune responses. YTHDF2 exerts context-dependent effects on tumor immunity. Left panel: YTHDF2 promotes tumor immune suppression by regulating IFN-γ/STAT1, CSF1, TRIM50, and AC026691.1 in M2 macrophage polarization; CD70, NF-κB, and Foxp3 in Treg cell activation and accumulation; and NF-κB, RXRα, BAMBI, and MYC in MDSC expansion, differentiation, and migration. Right panel: Conversely, YTHDF2 enhances anti-tumor immunity by promoting CD8^+^ T-cell activation and effector function through IKZF1/3, Cx3cl1, Gata3/Smad3, CD40L, and RIG-I, and by regulating NK cell survival, proliferation, chemotaxis, and maturation via Tardbp, STAT5, and SYTL1.

The tumor immune microenvironment (TIME) is composed of diverse cellular and molecular components and represents a key determinant of tumor initiation, progression, and therapeutic response (53–56). Its major constituents include: immune cells (e.g., dendritic cells, macrophages, myeloid-derived suppressor cells [MDSCs], natural killer [NK] cells, T cells, and B cells), which may either exert anti-tumor effects or be reprogrammed into tumor-promoting states; stromal cells (such as cancer-associated fibroblasts and endothelial cells) (57), which regulate immunity and angiogenesis via cytokine secretion and extracellular matrix remodeling; and soluble factors (e.g., cytokines, chemokines, and immune checkpoint molecules) (58), which orchestrate a dynamic balance between immune activation and suppression (53). Overall, the TIME constitutes a highly complex network, in which immunosuppressive features often enable tumor immune evasion. Thus, reprogramming the TIME has emerged as an important therapeutic strategy in immuno-oncology. In this review, we provide a comprehensive overview of the current progress on how the YTHDF protein family regulates tumor immunity (**Figures 2**, **3**).

Extensive studies have reported on the roles and mechanisms of YTHDF1 and YTHDF2 in tumor immune regulation. In colorectal cancer (CRC), YTHDF1 exerts immunosuppressive functions by remodeling the TIME (59). Its expression negatively correlates with interferon-γ–related gene signatures and CD8^+^ T-cell infiltration. Loss of Ythdf1 enhances anti-tumor immunity, reduces MDSCs, and increases cytotoxic T-cell responses, whereas overexpression promotes an immunosuppressive TIME. Mechanistically, YTHDF1 facilitates p65 translation, thereby activating the CXCL1–CXCR2 axis, driving MDSC recruitment, and suppressing CD8^+^ T-cell function. Functionally, inhibition of YTHDF1 not only sensitizes MSI-H CRC to PD-1 blockade but also overcomes the intrinsic resistance of MSS CRC, underscoring YTHDF1 as a potential immunotherapeutic target (59). Similarly, YTHDF1 is highly expressed in NASH-associated hepatocellular carcinoma (NASH-HCC) and promotes tumorigenesis. Liver-specific Ythdf1 overexpression in dietary models drives NASH-HCC development. Single-cell RNA sequencing and flow cytometry revealed that YTHDF1 fosters an immunosuppressive milieu by promoting MDSC accumulation and impairing CD8^+^ T-cell function. Mechanistically, YTHDF1 binds to m^6^A-modified EZH2 mRNA and enhances its translation (18). EZH2 subsequently induces IL-6 secretion, which mediates MDSC recruitment and activation, leading to CD8^+^ T-cell dysfunction. Functionally, Ythdf1 knockout or inhibition via LNP-siRNA significantly potentiates anti–PD-1 therapy, identifying YTHDF1 as a promising therapeutic target in NASH-HCC (18). Tumor-intrinsic YTHDF1 also promotes lysosomal gene translation, thereby accelerating MHC-I antigen degradation and impairing immune surveillance. In contrast, Ythdf1 deficiency reduces antigen degradation, converting “cold tumors” into “hot tumors” and substantially enhancing immune checkpoint inhibitor (ICI) efficacy. An exosome-mediated CRISPR/Cas9 delivery system has been developed to achieve *in vivo* YTHDF1 knockout, yielding potent anti-tumor effects. These findings not only elucidate the immune evasion mechanisms driven by YTHDF1 but also provide a solid experimental basis for its therapeutic targeting. YTHDF1 further suppresses anti-tumor immunity by regulating antigen presentation in dendritic cells (DCs). Ythdf1-deficient mice exhibit stronger antigen-specific CD8^+^ T-cell responses (14). Mechanistically, YTHDF1 recognizes m^6^A-modified lysosomal protease transcripts and promotes their translation, enhancing antigen degradation but impairing cross-presentation and T-cell activation (14). By contrast, Ythdf1-deficient DCs display reduced lysosomal degradation, improved cross-presentation, and enhanced anti-tumor immunity. Importantly, PD-L1 blockade is markedly more effective in the absence of Ythdf1. In gastric cancer (GC), YTHDF1 is overexpressed and correlates with poor prognosis (60). Functionally, Ythdf1 deletion suppresses GC cell proliferation and colony formation *in vitro*, and induces complete tumor regression in immunocompetent mice. Mechanistically, Ythdf1-deficient tumors exhibit enrichment of mature DCs (↑MHC-II, ↑IL-12), enhanced CD4^+^/CD8^+^ T-cell infiltration, and increased IFN-γ production, while activating the tumor cell IFN-γR1–JAK/STAT1 pathway, thereby heightening susceptibility to immune clearance. Moreover, Ythdf1 loss induces a durable “immune memory effect,” providing long-term protection against rechallenge with wild-type tumors (60).

The critical role of YTHDF2 in NK cell immunity was first identified. YTHDF2 is upregulated in NK cells upon cytokine stimulation, tumor challenge, or viral infection. Ythdf2 deficiency impairs NK cell anti-tumor and antiviral responses. Mechanistically, YTHDF2 regulates NK cell homing and Eomes expression to sustain homeostasis and terminal differentiation, while participating in a STAT5–YTHDF2 positive feedback loop that enhances IL-15–mediated survival and proliferation (61). Moreover, YTHDF2 targets Tardbp to regulate proliferation and viability, thereby strengthening NK effector function. In tumor-associated macrophages (TAMs), YTHDF2 is upregulated via the IL-10/STAT3 pathway and suppresses IFN-γ/STAT1 signaling, maintaining a pro-tumor phenotype. Loss of Ythdf2 reprograms TAMs toward an anti-tumor state, enhancing antigen cross-presentation and CD8^+^ T-cell immunity, thereby restraining tumor growth (62). TAM-targeted YTHDF2 knockdown using TLR9 agonist–conjugated siRNAs not only promotes TAM reprogramming but also augments anti–PD-L1 efficacy, suggesting that YTHDF2 is a promising immunotherapeutic target (62). Conversely, YTHDF2 sustains the immunosuppressive function of regulatory T cells (Tregs) specifically within the TME. While Treg-specific Ythdf2 loss does not disrupt peripheral immune homeostasis, it increases apoptosis and impairs suppressive activity of intratumoral Tregs, thereby slowing tumor progression. Mechanistically, tumor-derived TNF signaling induces YTHDF2 expression, which accelerates degradation of m^6^A-modified NF-κB negative regulators, maintaining NF-κB activation and Treg function (63). This highlights YTHDF2 as a critical regulator of intratumoral Tregs, whose selective targeting may enhance anti-tumor immunity while minimizing systemic autoimmunity. Similarly, YTHDF2 sustains the functionality of CD8^+^ T cells. Through an m^6^A-dependent mechanism, YTHDF2 promotes nascent RNA synthesis, stabilizes RNA homeostasis, and regulates mitochondrial function and chromatin remodeling, thereby preserving T-cell polyfunctionality and anti-tumor activity. Ythdf2 deficiency impairs T-cell function, accelerates tumor progression, and reduces responsiveness to PD-1 blockade (64). Mechanistically, YTHDF2 interacts with IKZF1/3 to maintain transcription of key target genes. Notably, treatment with lenalidomide, an IKZF1/3 inhibitor, partially restores immune function in Ythdf2-deficient T cells, underscoring YTHDF2 as a pivotal node linking epitranscriptomic and transcriptional regulation in T-cell immunity (64). Interestingly, radiotherapy (IR) induces YTHDF2 upregulation via NF-κB signaling, promoting MDSC expansion and immunosuppression, thereby driving radioresistance. Myeloid-specific Ythdf2 deletion blocks MDSC differentiation and infiltration, enhances anti-tumor immunity, and reverses radioresistance (15). Mechanistically, YTHDF2 degrades transcripts encoding NF-κB negative regulators, forming an IR–YTHDF2–NF-κB positive feedback loop. Pharmacological inhibition of YTHDF2 alleviates MDSC-mediated immunosuppression and markedly enhances radiotherapy efficacy, particularly when combined with anti–PD-L1, suggesting that YTHDF2 is a promising target to potentiate radio-immunotherapy (15). YTHDF2 also functions as a tumor-intrinsic regulator of immune evasion. Tumor-specific Ythdf2 loss suppresses tumor growth, prolongs survival, recruits macrophages via CX3CL1, reduces glycolysis, enhances CD8^+^ T-cell mitochondrial respiration, and promotes pro-inflammatory macrophage polarization and antigen presentation. IFN-γ induces YTHDF2 autophagic degradation, rendering tumors more vulnerable to CD8^+^ T-cell killing (65). Furthermore, a small molecule degrader of YTHDF2 exhibits robust anti-tumor activity as monotherapy and synergizes with PD-1/PD-L1 blockade, highlighting YTHDF2 as a potential therapeutic target. In hepatocellular carcinoma (HCC), YTHDF2 promotes tumor progression by orchestrating immune evasion and angiogenesis. Its expression is epigenetically activated by H3K4me3 and H3K27ac modifications and correlates with poor prognosis (66). Mechanistically, YTHDF2 recognizes m^6^A modifications in the 5′UTR of ETV5 mRNA, enhancing its translation. ETV5 subsequently activates transcription of PD-L1 and VEGFA, driving immune escape and angiogenesis. Functionally, Ythdf2 knockdown suppresses HCC progression, whereas overexpression accelerates tumorigenesis (66). Therapeutic targeting of YTHDF2 using siRNA aptamer/liposome complexes effectively inhibits immune evasion and angiogenesis, suggesting its potential as both a prognostic biomarker and therapeutic target. In Th9 cells, YTHDF2 suppresses differentiation by degrading m^6^A-modified Gata3 and Smad3 transcripts. Ythdf2 deficiency under IL-4 and TGF-β signaling promotes Th9 differentiation and enhances IL-9 and IL-21 secretion, thereby improving CD8^+^ T-cell and NK-cell infiltration and cytotoxicity, ultimately strengthening anti-tumor immunity (67). Moreover, YTHDF2 ablation enhances the activity of CAR-Th9 cells and improves their therapeutic efficacy, highlighting YTHDF2 as a potential target to boost Th9- and CAR-Th9–mediated immunotherapy (67). The seemingly contradictory roles of YTHDF1 and YTHDF2 in tumor immune regulation may arise from their context-dependent functions and cell-type specificity. YTHDF proteins exert distinct effects depending on the cellular environment, tumor type, and target mRNA repertoire. For instance, YTHDF2 may promote tumor progression by enhancing M2-like macrophage polarization, yet also restrain immunosuppression by modulating CD8^+^ T cell exhaustion or MDSC activity. Moreover, differences in experimental models, tumor microenvironmental cues, and m^6^A distribution patterns may further contribute to these divergent outcomes. Therefore, a more nuanced understanding of cell- and context-specific m^6^A dynamics is essential to reconcile these observations.

By contrast, the role of YTHDF3 in tumor immunity remains largely unexplored. Possible explanations include: (1) YTHDF3 is generally considered to act in concert with YTHDF1 and YTHDF2 in m^6^A regulation, lacking well-defined independent functions; (2) its molecular mechanisms are relatively redundant and difficult to distinguish from its paralogs in immune regulation; and (3) current research has predominantly focused on YTHDF1/2 given their prominent roles in tumor immunity and immunotherapy, leaving the immunological functions of YTHDF3 insufficiently investigated.

## 6 Therapeutic implications

As the roles of m^6^A modification and its readers in leukemia and tumor immunity are progressively elucidated, the YTHDF protein family has emerged as a novel class of therapeutic targets with considerable clinical potential. In leukemia, particularly AML, LSCs exhibit a much stronger dependency on YTHDF2 compared to normal HSCs (19). This differential requirement provides a selective therapeutic window for targeting YTHDF2. Experimental evidence has demonstrated that inhibition or knockdown of YTHDF2 markedly impairs the self-renewal and tumorigenic capacity of LSCs, while exerting only minimal long-term effects on HSC function (19). These findings highlight YTHDF2 as a promising and precise therapeutic target for the selective eradication of LSCs, offering a potential strategy to overcome relapse and drug resistance in leukemia. In the context of tumor immunity, YTHDF proteins have been shown to shape the immune microenvironment through multiple mechanisms. YTHDF1 in dendritic cells enhances the translation of genes involved in antigen processing and presentation, thereby influencing T-cell activation and tumor immunogenicity (13). Inhibition of YTHDF1 augments antitumor T-cell responses and improves sensitivity to ICIs. YTHDF2, by regulating the expression of inflammatory mediators and immunosuppressive molecules, contributes to immune tolerance and tumor immune evasion. Thus, targeting YTHDF2 may help reprogram the tumor immune microenvironment and enhance the efficacy of ICIs. Collectively, these findings suggest that inhibition of YTHDF1/2 holds synergistic potential when combined with current immunotherapeutic strategies (13). At present, drug development against YTHDF proteins remains in its infancy, but several strategies have emerged: Small-molecule inhibitors: Designed to disrupt the interaction between YTHDF RNA-binding domains and m^6^A-modified RNA. PROTAC technology: Exploiting the ubiquitin–proteasome system to selectively degrade YTHDF proteins (23, 68), thereby overcoming the specificity limitations of conventional inhibitors. RNA-targeting approaches: Employing antisense oligonucleotides or RNA interference to target specific YTHDF-dependent transcripts and block their oncogenic functions. Given the multi-pathway drivers and strong drug resistance frequently observed in leukemia and solid tumors, targeting YTHDF proteins alone may not achieve durable therapeutic efficacy. Consequently, increasing efforts are focused on combination strategies, including: With chemotherapy: Targeting YTHDF2 to eliminate LSCs and reduce relapse risk. Overall, the YTHDF proteins represent highly attractive therapeutic candidates in both leukemia and tumor immunity. Future research should prioritize selective modulation of YTHDF protein activity, delineation of their context-dependent mechanisms, and optimization of combination strategies with existing therapies, with the ultimate goal of translating these mechanistic insights into clinically applicable treatments.

Given their pivotal roles in modulating tumor immune responses, targeting the YTHDF family offers a promising strategy to enhance the efficacy of immunotherapy. Modulation of YTHDF1 or YTHDF2 activity could reshape the tumor immune microenvironment by influencing antigen presentation, cytokine production, and immune cell infiltration. For example, inhibiting YTHDF1 has been shown to potentiate the response to immune checkpoint blockade by improving antigen cross-presentation, while fine-tuning YTHDF2 function may alleviate immune exhaustion or reduce MDSC-mediated suppression. Therefore, combining YTHDF-targeted approaches with existing immunotherapies, such as PD-1/PD-L1 or CTLA-4 blockade, could synergistically overcome tumor immune resistance. Future studies should focus on identifying specific modulators of YTHDF proteins and clarifying their cell-specific effects to translate these findings into clinical applications.

## 7 Limitations and future directions

Despite substantial progress in understanding the biological functions of YTHDF proteins in hematopoiesis, leukemogenesis, and cancer immunity, several important limitations remain. First, the field still lacks a systematic framework to explain the context-dependent and interdependent functions of the YTHDF family. Different studies often report contradictory phenotypes, particularly for YTHDF1, which has been described as either oncogenic or tumor-suppressive depending on the cancer type, experimental system, or microenvironmental cues (69, 70). These discrepancies highlight the need for integrated analyses that consider cellular identity, developmental stage, metabolic state, and immune context. Second, due to the high structural similarity among YTHDF1, YTHDF2, and YTHDF3, achieving selective inhibition of individual family members remains a major challenge. Most currently available targeting strategies simultaneously affect all three proteins, making it difficult to dissect member-specific functions and increasing the risk of off-target toxicity in therapeutic applications (71). A third limitation lies in the incomplete mechanistic understanding of the seemingly paradoxical roles of YTHDF2 in immune regulation. YTHDF2 has been reported to enhance the cytotoxicity of NK cells and CD8^+^ T cells while simultaneously maintaining the suppressive functions of regulatory or myeloid-derived immunosuppressive cells (71). The molecular basis of this duality remains unresolved and likely reflects cell type–specific mRNA substrates, distinct post-translational modifications, and microenvironment-dependent regulatory networks. Finally, YTHDF3 remains understudied in tumor immunity. It is unclear whether its limited characterization reflects a relatively minor biological role or whether its functions are masked by strong interdependence with YTHDF1 and YTHDF2. Moreover, additional mechanistic layers-including chromatin-associated RNA regulation, chromatin accessibility, 3D genome organization, liquid–liquid phase separation (LLPS), and potential m6A-independent functions-have only begun to be explored and represent important blind spots in current research.

Future studies should focus on elucidating the mechanistic basis of the divergent immune phenotypes mediated by YTHDF2. Dissecting its cell type–specific mRNA targets, dynamic m6A landscapes, and interactions with distinct signaling pathways will be essential for reconciling its pro-cytotoxic versus immunosuppressive roles. Another important direction is to determine how tumor heterogeneity, mutational background, and microenvironmental factors influence the functions of the YTHDF family. Integrating single-cell multi-omics with *in vivo* genetic models will help clarify how YTHDF proteins operate across different leukemic and immune cell states. In addition, expanding research into chromatin-related roles of YTHDF proteins-including their involvement in chromatin-associated RNAs, enhancer regulation, and 3D genome architecture-may uncover previously unrecognized layers of epigenetic control. Understanding the biophysical properties of YTHDF proteins is also a key priority. Whether LLPS is strictly dependent on m6A recognition, and whether YTHDF proteins possess functions independent of m6A binding, remain open questions. These issues can be addressed through biochemical reconstitution, structural analyses, and mutant-rescue approaches *in vivo*. Advancing selective targeting strategies is equally important. Structural and proteomic characterization of non-conserved domains, intrinsically disordered regions, or unique protein–protein interaction interfaces may enable the development of member-specific inhibitors. Coupling such inhibitors with cell-type-directed delivery systems or targeted protein degradation technologies may further improve specificity and therapeutic feasibility. Collectively, addressing these limitations will provide deeper mechanistic insight into how YTHDF proteins integrate RNA metabolism, leukemic progression, and immune regulation. Moreover, defining the contextual determinants of YTHDF function and developing selective targeting strategies will be essential steps toward translating m6A-reader biology into precision therapies for hematologic malignancies and cancer immunotherapy.

## 8 Conclusion

As one of the most important families of m^6^A readers, YTHDF proteins play pivotal roles in hematopoiesis, leukemogenesis, and tumor immune regulation. By precisely modulating mRNA stability and translational efficiency, they govern the fate of HSCs and LSCs, while profoundly influencing immune cell functional states and shaping the tumor immune microenvironment. Recent studies have revealed that YTHDF proteins are not only central executors of epitranscriptomic regulation but also critical hubs linking hematopoietic homeostasis, malignant transformation, and immune evasion. From a therapeutic perspective, the strong dependency of LSCs on YTHDF2 highlights its potential as a selective target, while the roles of YTHDF1/2 in immune regulation offer new avenues for improving immune checkpoint therapy. With the rapid development of small-molecule inhibitors, PROTACs, and RNA-targeting strategies, YTHDF proteins are gradually transitioning from basic research toward clinical applications. In summary, unraveling the functional specificity and context dependency of YTHDF proteins will not only refine our understanding of hematopoietic and immune regulation but also provide valuable opportunities for the development of novel therapeutic strategies. Looking forward, YTHDF proteins are poised to serve as a crucial bridge between epitranscriptomic research and precision medicine, opening new directions for leukemia treatment and cancer immunotherapy.
